# A Corticothalamic Circuit Trades off Speed for Safety during Decision-Making under Motivational Conflict

**DOI:** 10.1523/JNEUROSCI.0088-22.2022

**Published:** 2022-04-20

**Authors:** Eun A. Choi, Medina Husić, E. Zayra Millan, Sophia Gilchrist, John M. Power, Philip Jean-Richard dit Bressel, Gavan P. McNally

**Affiliations:** ^1^School of Psychology, UNSW Sydney, 2052 New South Wales, Australia; ^2^Cognitive and Systems Neuroscience Group, SILS Center for Neuroscience, University of Amsterdam, Amsterdam, 1090 GE, The Netherlands; ^3^Translational Neuroscience Facility, School of Medical Sciences, UNSW Sydney, 2052 New South Wales, Australia

**Keywords:** impulsivity, paraventricular thalamus, punishment, reward, salience, ventral hippocampus

## Abstract

Decisions to act while pursuing goals in the presence of danger must be made quickly but safely. Premature decisions risk injury or death, whereas postponing decisions risk goal loss. Here we show how mice resolve these competing demands. Using microstructural behavioral analyses, we identified the spatiotemporal dynamics of approach–avoidance decisions under motivational conflict in male mice. Then we used cognitive modeling to show that these dynamics reflect the speeded decision-making mechanisms used by humans and nonhuman primates, with mice trading off decision speed for safety of choice when danger loomed. Using calcium imaging in paraventricular thalamus and optogenetic inhibition of the prelimbic cortex to paraventricular thalamus pathway, we show that this speed-safety trade off occurs because increases in paraventricular thalamus activity increase decision caution, thereby increasing approach–avoid decision times in the presence of danger. Our findings demonstrate that a discrete brain circuit involving the paraventricular thalamus and its prefrontal input adjusts decision caution during motivational conflict, trading off decision speed for decision safety when danger is close. We identify the corticothalamic pathway as central to cognitive control during decision-making under conflict.

**SIGNIFICANCE STATEMENT** Foraging animals balance the need to seek food and energy against the conflicting needs to avoid injury and predation. This competition is fundamental to survival but rarely has a stable, correct solution. Here we show that approach–avoid decisions under motivational conflict involve strategic adjustments in decision caution controlled via a top-down corticothalamic pathway from the prelimbic cortex to the paraventricular thalamus. We identify a novel corticothalamic mechanism for cognitive control that is applicable across a range of motivated behaviors and mark paraventricular thalamus and its prefrontal cortical input as targets to remediate the deficits in decision caution characteristic of unsafe and impulsive choices.

## Introduction

Foraging animals balance the need to seek food and energy against the conflicting needs to avoid injury and predation. Resolving this conflict is fundamental to survival, but there is rarely a stable, single solution. Instead, appropriate solutions vary across space and time (diurnal, seasonal) as well as internal states (hunger, thirst). These decisions thus involve compiling sensory information about the world with knowledge about reward or danger to support adaptive behavior. These decisions must be made quickly and safely. They require balancing the competing demands of speed in decision-making with safety of choice. Premature decisions risk injury or death, whereas failures to decide in a timely manner risk goal loss. Although much is known about the brain mechanisms of danger and reward (Mobbs et al., 2020), the mechanisms for this decision-making under motivational conflict are poorly understood.

The prelimbic cortex (PL) is well established in approach–avoid decision-making (Verharen et al., 2019; Kyriazi et al., 2020; Fernandez-Leon et al., 2022). For example, activity of PL units during conflict reflects a variety of task variables (valence, trial identity, response) (Kyriazi et al., 2020) that contribute to individual approach–avoid decisions (Fernandez-Leon et al., 2022) and specifically control approach behaviors under threat (Verharen et al., 2019, 2020). These roles are due, at least in part, to prefrontal interactions with amygdala and striatum (Verharen et al., 2019, 2020; Kyriazi et al., 2020). The ventral hippocampus, and in particular the ventral subiculum (vSub), is also well established in approach–avoidance conflict (Gray, 1982; O'Neil et al., 2015; Ito and Lee, 2016; Schumacher et al., 2016; Gray and McNaughton, 2000; Çavdaroğlu et al., 2021). For example, human neuroimaging studies show hippocampal recruitment during approach–avoidance conflict (Bach et al., 2014; O'Neil et al., 2015), and pharmacological (Nguyen et al., 2015; Schumacher et al., 2016) or chemogenetic (Marchant et al., 2016) manipulation of vSub in rodents alters decision-making under conflict. Finally, recent findings implicate the paraventricular thalamus (PVT) in approach–avoidance conflict (Choi and McNally, 2017; Zhu et al., 2018; Choi et al., 2019; McNally, 2021), showing that, like vSub, PVT neurons are strongly recruited by both reward and danger and that PVT silencing disrupts behavior during, but not in the absence of, motivational conflict.

Nonetheless, there are at least three outstanding questions about the roles of these regions in motivational conflict. First, although the behavioral designs used in many of these studies clearly identify roles in approach–avoidance conflict, these designs typically do not isolate the discrete psychological mechanisms of approach–avoid decisions. So how these regions relate to specific approach–avoid decision-making mechanics is poorly understood. Second, although both PVT and vSub are implicated in behavioral responses under approach–avoid conflict, the different roles of these regions are not known because they have not been directly compared. Finally, the circuit mechanisms for these roles in approach–avoidance conflict are poorly understood. For example, PL is a major source of excitatory glutamatergic inputs driving PVT neuronal activity (Vertes, 2002; Li and Kirouac, 2012; Otis et al., 2019), but the role of this corticothalamic pathway in motivational conflict is unknown.

Here we used a well-established approach–avoidance task to address these issues. We trained mice to approach a goal with conflicting values of reward and punishment and studied the microstructure of behavior under this conflict. Then we used formal cognitive modeling to identify the latent mechanisms of choice under conflict. We next examined how the spatiotemporal activity dynamics of two brain regions (PVT and vSub) related to these approach–avoidance choice mechanics. Finally, we used circuit-specific optogenetic inhibition to establish a causal role for the PVT and its PL input in decision-making under motivational conflict. Our findings show that a discrete brain circuit involving the PVT and its prefrontal input dynamically adjusts decision caution when making choices under motivational conflict, trading off decision speed for decision safety when danger looms.

## Materials and Methods

### Subjects

Male C57BL/6J mice (Australian Resources Center) 8-10 weeks of age were used. They were housed in ventilated racks, in groups of 2-4, on corn cob bedding in a climate-controlled colony room maintained on 12:12 h light/dark cycle (0700 lights on). They had free access to food (Gordon's Mice Chow) and water until 2 d before commencement of behavioral training when they received 30 min of access to water each day for the remainder of the experiment. The animals were in good health. Mice will learn the task when not thirsty, but we adopted this manipulation because rodents often forage (and hence face and solve approach–avoid conflict) in deprivation states.

Experiments were approved by the UNSW Animal Care and Ethics Committee and performed in accordance with the Animal Research Act 1985 (NSW), under both ARRIVE guidelines and the National Health and Medical Research Council Code for the Care and Use of Animals for Scientific Purposes in Australia (2013).

### Surgeries and viral injections

Mice were deeply anesthetized with 5% isoflurane in oxygen-enriched air after subcutaneous injection of 5 mg/kg carprofen (Rimadyl, Zoetis) and then fixed into a stereotaxic alignment instrument (Model 1900, Kopf Instruments). During surgery, mice were maintained on 1%-2.5% isoflurane. Before the scalp incision, a local injection of 0.1 ml Marcaine (0.5%) was made subcutaneously at the incision site. Ophthalmic gel (Viscotears, Alcon) was applied to avoid eye drying. Mice received injections of antibiotic (Duplocillin, 0.15 ml/kg of body weight subcutaneously) immediately after surgery.

Adeno-associated viruses (AAVs, 0.5 μl) were delivered using a 33-gauge conical tipped microinfusion syringe (SGE Analytical Science) connected to a UMP3 with SYS4 Micro-controller microinjection system (World Precision Instruments). vSub coordinates were as follows: 3.15 mm posterior, 2.75 mm lateral, and 4.7 mm below bregma. PVT coordinates were as follows: 1.4 mm posterior, 0 mm lateral, and 3.15-3.35 mm below bregma. PL coordinates were as follows: 1.94 mm anterior, 0.35 mm lateral, and 2.2-2.6 mm below bregma.

pAAV-CamKII-eNpHR 3.0-EYFP was a gift from Karl Deisseroth (Addgene viral prep #26972-AAV5; http://n2t.net/addgene:26972; RRID:Addgene_26972; 4.9 × 10^12^ vp/ml). pGP-AAV-syn-jGCaMP7f-WPRE was a gift from Douglas Kim & GENIE Project (Addgene viral prep #104488-AAV9; http://n2t.net/addgene:104488; RRID:Addgene_104488; 2.6 × 10^13^ vp/ml). AAV5-hSyn-eYFP (4 × 10^12^ vp/ml) was obtained from UNC Vector Core (University of North Carolina, Chapel Hill, NC).

### Behavioral procedures

Experiment 1 (*N* = 8) studied the microstructural analyses of behavior under motivational conflict. They were placed into a linear track (120 cm [l] × 15 cm [w] × 40 cm [h]) constructed of Perspex. The first 22 cm was a Start box, constructed from gray and white Perspex walls and a Perspex floor. The Start box was separated from the remainder of the track by a removable Perspex door. The next 88 cm was the track proper constructed from white Perspex walls and a white Perspex floor. The Goal box was the last 10 cm and was constructed from gray Perspex walls and a stainless-steel grid floor. The Goal box contained a stainless-steel receptacle extending 3 cm from the end wall for delivery of liquid reward. A rail ran above the track to which all fiber optic patch cables were attached.

For training, there were four trials a day for 4 d. Each trial commenced with removal of the door between the Start box and the track; 10 μl of 8% sucrose was available from the receptacle in the Goal box. The trial ended after 2 min or after mice had consumed the sucrose. At the end of the trial, mice were returned to the Start box for 30 s until the start of the next trial.

For conflict, the same procedures were used but the grid floor in the Goal box was electrified using a 0.05, 0.075, or 0.1 mA current. Mice received 1 d of training at 0.05 mA, 2 d at 0.075 mA, and 2 d at 0.1 mA.

Mice were tracked (30 fps) via webcam (Logitech C920, 1080p) connected to a computer running EthoVision XT 10 (Noldus Information Technology). Ethovision tracked the *x* and *y* coordinates of the animal's center. From these coordinates, the following variables were computed: time spent in Start box, linear track, Goal box; distance from the goal; and velocity of the center-point of the animal. These data were imported into MATLAB R2018b (The MathWorks) for further analysis.

### Fiber photometry

Experiment 2 (*N* = 12) used fiber photometry to study PVT and vSub during conflict. We expressed an AAV encoding gCaMP7f in the PVT and implanted a fiber optic cannula above the expression site. After reward training (see above), mice received 1 d of conflict training with 0.05 mA footshock. They were tested the following day with 0.05 mA footshock. During this test, mice were tethered to a single fiber optic patch cable attached to a rail that ran above the linear track, providing unhindered motion. Recordings were performed using Fiber Photometry Systems from Doric Lenses and Tucker Davis Technologies (RZ5P, Synapse). Excitation lights (465 nm Ca^2+-^dependent and 405 nm isosbestic control signal) emitted from LEDs (LEDC1-B_FC, LEDC1-405_FC; Doric Lenses), controlled via dual-channel programmable LED drivers (LEDD_4, Doric Lenses), were channeled into 0.39 NA, Ø400 μm core multimode prebleached patch cables via a Doric Dual Fluorescence Mini Cube (FMC2, Doric Lenses). Light intensity at the tip of the patch was maintained at 10-30 µW across sessions. Ca^2+^ and isosbestic fluorescence were measured using femtowatt photoreceivers (Newport, 2151). Synapse software controlled and modulated excitation lights (465 nm: 209 Hz; 405 nm: 331 Hz), and demodulated and low-pass filtered (3 Hz) transduced fluorescence signals in real time via the RZ5P. Synapse/RZ5P also received timestamping TTL signals from Ethovision.

### Electrophysiology

Experiment 3 (*N* = 6) used electrophysiology. We expressed an AAV encoding the eNpHR3.0 in the PL using the procedures described above and made whole-cell recordings from PVT neurons while electrically evoking activity in the PL→PVT pathway.

Mice were anesthetized with 5% isoflurane gas and decapitated. Brains were extracted, and sagittal or coronal slices (300 µm) containing the PVT were prepared with a Vibratome (Model VT1200, Leica Microsystems). Slices were cut in ice-cold NMDG-modified aCSF containing the following (in mm): 95 N-methyl-D-glucamine, 2.5 KCl, 30 NaHCO_3_, 1.2 NaH_2_PO_4_, 20 HEPES, 30 glucose, 5 sodium ascorbate, 2 thiourea, 3 sodium pyruvate, 10 N-acetyl L-cysteine, 0.5 CaCl_2_, 10 MgSO_4_ (pH adjusted to 7.3-7.4 with HCl and osmolality between 300 and 310 mOsm). Slices were maintained at 30°C for 10 min in the low-calcium NMDG-modified solution before being transferred to a Braincubator (#BR26021976, Payo Scientific) and continuously perfused with a HEPES-modified aCSF solution containing the following (in mm): 95 sodium chloride, 2.5 KCl, 30 NaHCO_3_, 1.2 NaH_2_PO_4_, 15 HEPES, 20 glucose, 5 sodium ascorbate, 2 thiourea, 3 sodium pyruvate, 10 N-acetyl L-cysteine, 2 CaCl_2_, 2 MgSO_4_ (pH adjusted to 7.3-7.4 with NaOH and osmolality between 300 and 310 most) for up to 24 h. All solutions were oxygenated (95% O_2_, 5% CO_2_).

Slices were transferred to a recording chamber and perfused with oxygenated standard aCSF containing the following (in mm): 124 sodium chloride, 3 KCl, 26 NaHCO_3_, 1.2 NaH_2_PO_4_, 10 glucose, 2.5 CaCl_2_, 1.3 MgSO_4_. Slices were heated to 30°C. Neurons in the PVT were targeted, and projecting terminals from the PLPFC were visualized with the aid of a wide-field microscope (Zeiss Axio Examiner D1) equipped with 20× water immersion objective (1.0 NA), LED fluorescence illumination system (pE-2, CoolLED) and EMCCD camera (iXon+, Andor Technology). Whole-cell patch-clamp recordings were performed with low-resistance (3-5 mΩ) pipettes made from borosilicate glass (GC120TF-4; Warner Instruments) using a two-stage vertical puller (PC-10; Narishige). Patch pipettes contained a Cs^+^-based internal solution containing the following (in mm): 110 Cs methanesulfonate, 8 sodium chloride, 10 HEPES, 2 Mg_2_-ATP, 0.3 Na_3_-GTP, 0.1 spermine, 7 phosphocreatine, 10 EGTA, 5 QX314 (pH adjusted to 7.3-7.4 and osmolality to 280-300 mOsm using CsOH); 0.1 mm picrotoxin (from 400 mm stock in DMSO) was bath-applied to all slices to prevent GABAergic currents.

Electrical stimulation was provided by a Constant Voltage Isolated Stimulator (Digitimer), delivered through a borosilicate glass pipette filled with standard aCSF and with the tip broken to allow greater electrical access. The stimulating electrode was positioned above projecting fluorescent PL→PVT fibers around the PVT. Stimulator and cell locations were determined from live slices with the aid of a wide-field microscope (Zeiss Axio Examiner D1) equipped with 2.5× (0.075 NA) and 5× (0.16 NA) objectives. eYFP was visualized with a 470/40 excitation filter, 525/50 emission filter, and 495 dichroic filter. Optogenetic inhibition of these fibers was simultaneously delivered through the 20× objective. Electrophysiological recordings were amplified using a Multiclamp 700B amplifier (Molecular Devices) filtered at 6 kHz and digitized at 20 kHz with a Digidata1440A (Molecular Devices). Recordings were controlled and analyzed offline using Axograph (Axograph). The locations of all recorded cells were mapped according to the Mouse Brain Atlas (Paxinos and Watson, 2019). The liquid junction potential (∼9 mV) was not compensated for.

Electrically evoked currents were investigated with 99 V pulses (between 120 and 160 µs) delivered in 10 s intervals while holding the cell at −70 mV. Optogenetic inhibition occurred every second episode, to allow comparison between the inhibited and the noninhibited electrically stimulated postsynaptic currents. The protocol was repeated 100 times.

### Optogenetics

Experiment 4 (*N* = 12) used optogenetics to study the causal role of the PL→PVT pathway in behavior under conflict. We expressed an AAV encoding the eNpHR3.0 or eYFP in the PL using the procedures described above and implanted a fiber optic cannula above the PVT to inhibit the PL→PVT pathway. After reward training (see above), mice received 1 d of conflict training with 0.05 mA footshock (see above). They were tested the following 2 d under conflict (0.05 mA). During both tests, mice were tethered to a single fiber optic patch cable attached to the rail that ran above the linear track, providing unhindered motion, and which connected to 625 nm LEDs (Doric Lenses) controlled by Ethovision. During one test (Off), there was no optical stimulation. During a second test (On), continuous 625 nm optical stimulation (10-12 mw/mm^2^ measured at the tip of an unimplanted fiber) was delivered only when mice were located within 8 cm of the goal.

### Histology and immunohistochemistry

Mice from Experiments 1, 2, and 4 were anesthetized with intraperitoneal injections of sodium pentobarbital (100 mg/kg) and perfused with 0.9% saline solution containing 1% sodium nitrate and heparin (5000 IU/ml), followed by PB solution (0.1 m) with 4% PFA. Brains were extracted, incubated in 20% sucrose solution for cryoprotection, sliced coronally (40 μm) using a cryostat, and stored in PB solution with 0.1% sodium azide at 4°C.

Fiber placements and AAV expression were determined via immunohistochemistry and native fluorescence. An eGFP antibody was used to detect AAV-expressing cells. Sections were washed with PB solution (0.1 m PB, pH 7.4), 50% ethanol, 50% ethanol with 3% hydrogen peroxidase, and then 5% normal horse serum (NHS) in PB for 30 min each. The sections were then incubated for 48 h in chicken antiserum against eGFP (1:2000; Invitrogen, catalog #A10262 RRID:AB_2534023) in 2% NHS-PBTx (0.2% Triton X-10 in PB) with 0.1% sodium azide at room temperature. After washing in PB, sections were incubated in biotinylated donkey anti-chicken (1:2000; Jackson ImmunoResearch Laboratories; catalog #703-035-155 RRID:AB_10015283 24 h at room temperature) in 2% NHS-PBTx. The sections were washed and incubated in avidin-biotinylated HRP complex (Vector Elite kit: avidin and biotin, each 6 µl/ml; Vector Laboratories) in PBTx. Then, the sections were washed in PB and 0.1 m acetate buffer (pH 6.0) and incubated (15 min) in a DAB solution containing 0.1% 3,3-diaminobenzidine, 0.8% D-glucose, and 0.016% ammonium chloride. Immunoreactivity was catalyzed by the addition of 0.2 µl/ml glucose oxidase (24 mg/ml, 307 U/mg; Sigma-Aldrich). Tissue was washed with PB and mounted onto gelatinized slides. Slides were left to dry and then cover-slipped. AAV expression and cannula placements were verified using light and fluorescent microscopy. Animals were excluded from analyses if fiber tip and AAV expression could not be confirmed as colocalized.

### Linear ballistic accumulator (LBA) modelling

The LBA is an exemplar accumulation model of decision-making and the simplest, complete model of choice with an analytical solution. Choice in the LBA depends on five parameters (*v*, *s*, *A*, *b*, *t_0_*,) where *v* is the accumulation rate for each response option (sampled on each trial from a normal distribution with mean *v*_i_ and SD *s*_i_), *s* is between-trial variation in *v*, *A* is the starting point of the accumulation process (sampled on each trial from a uniform distribution), *b* is the amount of evidence needed to make a response, and *t_0_* is the nondecision time (perceptual and motor processing) (Brown and Heathcote, 2008). Inferences about decision-making processes from LBA are similar to other sequential sampling models with the key advantage that the LBA can scale to any number of response options.

Following Annis et al. (2017), we set *s* to a constant value (1) and assumed accumulation rate priors for each response were truncated normal distributions (mean = 2, SD = 1), a uniform prior nondecision time (0, 1), maximum starting evidence for *A* was a truncated normal distribution (mean 0.5, SD = 1), and determined a relative threshold, *k*, from a truncated normal distribution (mean 0.5, SD = 1), from which we could derive *b* as *k* + *A*. Response caution was then defined as *b* – *A*/2. We used a Hamiltonian Markov Chain Monte Carlo (HMCMC) algorithm (warmup = 1000; iteration = 2000; thinning = 1; δ = 0.8) to obtain posterior distributions. Three chains were run to evaluate convergence with a Gelman-Rubin's criteria of R ^ < 1.1 (Gelman and Rubin, 1992) and an effective sample size (Neff) > 100 (Gelman et al., 2013). All analyses were performed using R (version 4.0.2) (R Development Core Team, 2017) via R Studio (version 1.3.959) and the RStan package (Hoffman and Gelman, 2014; Stan Development Team, 2015).

### Experimental design and statistical analyses

Data in figures are represented as individual data points overlaid with mean ± SEM unless otherwise stated. The only criteria for inclusion in final analyses were correct AAV and/or fiber placements determined after histology. Group numbers for each experiment are indicated in Results. Inferential statistics were based on within-subjects *t* tests or Wilcoxon-signed rank tests, ANOVA, curve-fitting, and multiple regression. All analyses were conducted using GraphPad Prism version 8.4.2, MATLAB, SPSS 25, and the Psy statistical package.

Before locomotor behavior was analyzed, frames with no or poor tracking were identified and replaced using linear interpolation. Then, for each frame, a custom script used the *x*-center coordinates to determine whether the mouse was moving (≥1.5 cm uninterrupted) toward the Start box, the Goal box, or paused (defined as no movement for ≥90 ms followed by movement of ≥1.5 cm). Only pauses that occurred on the track (i.e., outside the start or Goal box) were considered for analysis and modeling.

Electrophysiological analyses were performed using Axograph (Axograph). Data were excluded from analysis if >500 pA was required to maintain the neuron at –65 mV. Passive properties, such as input resistance and membrane capacitance, were calculated from injection of small, hyperpolarizing pulses (–5 mV) in voltage clamp at –65 mV. Membrane time constant was determined by fitting an exponential to the voltage deflection caused by a small hyperpolarizing current (–5 to –20 pA; 600 ms).

For fiber photometry, Ca^2+^-dependent (465 nm-related) and isosbestic (405 nm-related) signals and event timestamps were extracted into MATLAB. The isosbestic signal was linearly regressed onto the Ca^2+^-dependent signal to create a fitted isosbestic signal, and a normalized fluorescence change score (dF/F) was calculated using the standard formula: (Ca^2+^-dependent signal – fitted isosbestic)/fitted isosbestic. Phasic neural activity change around pauses was determined by collating dF/F around pause events (−5 s to 5 s around pause onset or offset, baselined to −5 s to −2.5 s before event). To determine significance of activity change, 95% CIs around activity kernels were derived via bootstrapping (Jean Richard dit Bressel et al., 2020). Bootstrapped means were obtained by randomly resampling from subject mean waveforms with replacement (1000 iterations). CI limits were derived from 2.5 and 97.5 percentiles of bootstrap distribution, expanded by a factor of √(*n*/(*n* – 1)). A significant transient was identified as a period that CI limits did not contain 0 (baseline) for at least 1/3 s (low-pass filter window). To assess the relationship between neural activity and LBA parameters, mean dF/F during pauses at the three runway locations were calculated per subject and correlated against estimates of LBA parameters per subject for these locations.

## Results

### Microstructure of behavior under conflict

**Figure 1.**
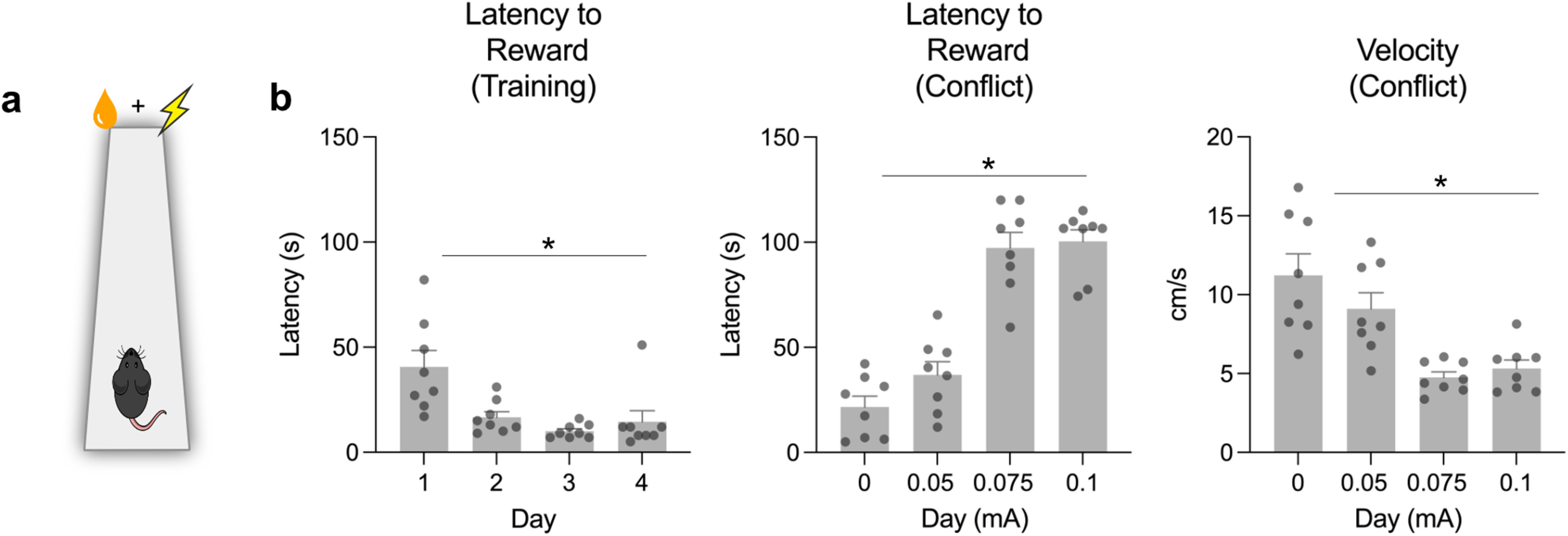
***a***, Conflict paradigm. ***b***, Mean and SEM behavioral measure of conflict. Latencies to obtain reward reduced across training. Conflict increased the time taken to obtain reward, decreased running speeds, increased time in the Start box, time on the runway, and the number of transitions between the Start box and runway. *p < 0.05.

In Experiment 1, we trained thirsty mice (*n* = 8) to run a linear track to obtain liquid sugar reward from a Goal box; and as expected, we found that latencies to retrieve the reward decreased across training (*F*_(3,21)_ = 11.56, *p* = 0.002) (Fig. 1*a*,*b*). Conflict was then introduced by electrifying the floor of the Goal box, with current intensity increasing across days (0.05, 0.075, 0.1 mA). Under these conditions, mice learn reward and danger (Hull, 1938; Miller, 1944), which are retrieved via hippocampal awake replay (Wu et al., 2017) to support approach or avoidance decisions across the track. As expected, running speeds decreased (*F*_(3,21)_ = 8.431, *p* = 0.0001), whereas time taken to obtain reward increased (*F*_(3,21)_ = 75.21, *p* < 0.0001) across days of conflict training.

**Figure 2.**
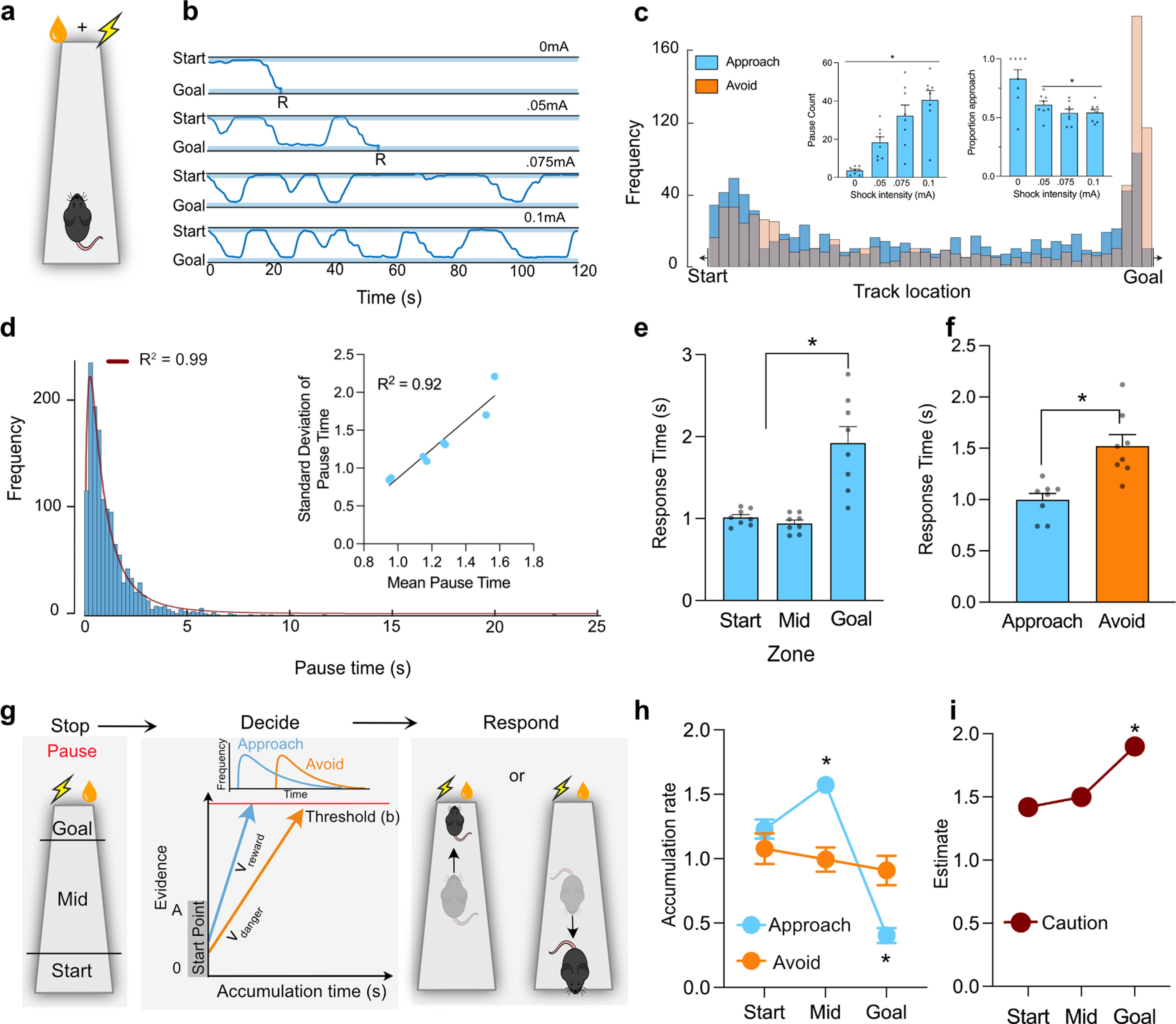
Spatiotemporal properties of decision-making under motivational conflict. ***a***, Conflict paradigm (*n* = 8). ***b***, Example trajectories from no conflict (0 mA) and conflict tests. R indicates consumption of reward. ***c***, Spatial distribution of pauses. Inset, Mean and SEM (*n* = 8) pause counts and proportion approach decisions. ***d***, Frequency distribution of pause durations and log-normal fit. Inset, Correlation of mean pause times and SDs. ***e***, Mean and SEM RTs for the three track zones. ***f***, Mean and SEM RTs for approach versus avoid decisions. ***g***, The LBA model of choice. Salience (V) of reward and danger accumulates separately to an evidence threshold (b) in a winner-takes-all process. ***h***, Mean and SEM accumulation rates for approach and avoid decisions across the zones. ***i***, Mean and SEM response caution across the zones. *p < 0.05. For an example of Hamiltonian Markov Chain Monte Carlo modeling, see Extended Data Figure 2-1.

Visualization of individual trajectories showed that, in the absence of conflict, mice would proceed directly along the track to the goal and consume the reward (Fig. 2*b*). However, under conflict, mice exhibited bistable oscillations between the start and the end of the track (Fig. 2*b*). These oscillations were interrupted by pauses, ranging from milliseconds to seconds in duration. During these pauses, mice would rear, show lateral head scanning movements, and investigative sniffing (Wu et al., 2017; Thompson et al., 2018). These pauses were observed across the track, but they peaked at the start of the track and at the end of the track just before the goal (Fig. 2*c*), consistent with pauses reflecting active sampling of the environment to prompt individual approach or avoid decisions (Redish, 2016; Bach and Dayan, 2017; Mobbs et al., 2020). Pauses increased as shock intensity increased (*F*_(1,7)_ = 47.957, *p* = 0.0001) (Fig. 2*c*, inset).

10.1523/JNEUROSCI.0088-22.2022.f2-1 Figure 2-1 Bayesian parameter estimation of LBA via Hamiltonian Markov Chain Monte Carlo. a) Autocorrelation functions for samples returned by Stan. Autocorrelations dropped to zero at around lags of 10, indicating that the sampler efficiently explored the posteriors for each parameter. b) Samples from each chain as a function of iteration showing strong central tendencies, remaining around constant values, with strong overlap between chains, indicating convergence to the posterior distribution. c) Posterior predictive check indicating good fit between the model predictions (line) and the observed (histograms) decision times. Warmup = 1000; iteration = 2000; thinning = 1; delta = 0.8. Three chains were run to evaluate convergence with a Gelman-Rubin's criteria of R ^ < 1.1 and an effective sample size (N_eff_) > 100. Download Figure 2-1, TIF file.

By identifying each pause, we could ask how each approach–avoid decision was resolved. There were notable spatial biases in decision outcomes. Approach decisions dominated across most of the track but were replaced by avoidance decisions closer to the goal (Fig. 2*c*). In the absence of danger, there were few decisions, and these were resolved in favor of approach. In the presence of danger, the frequency of decision-making increased and the approach bias was lost (Fig. 2*c*, inset: 0 mA vs shock, *F*_(1,17)_ = 10.265, *p* = 0.015).

Response times (RTs) to choose approach or avoid after pause onset were measured because RTs are the most robust and widely used measure of decision-making efficiency (Laming, 1968). RTs were positively skewed, log-normal functions (*R*^2^ = 0.99, *F*_(1,122)_ = 1312, *p* < 0.0001) with linear coefficients of variation (*R*^2^ = 0.93, *F*_(1,6)_ = 80.59, *p* < 0.0001) (Fig. 2*d*). *k*-means clustering identified distinct patterns of decision-making across the track. Pauses within 5 cm bins were collated, and median location, total number of pauses, median pause duration, % approach versus avoid decisions were used as inputs. Silhouette values for three clusters were positive (mean = 0.694, minimum = 0.243), indicating location thresholds at 5 cm from goal and 15 cm from start within the linear track, generating three zones of approach–avoid decision-making on the track itself (i.e., excluding start and Goal box): start zone (0-15 cm), mid zone (15-83 cm), and goal zone (83-88 cm). Approach–avoid decisions at the goal zone were most difficult, taking the longest time (main effect location: *F*_(2,14)_ = 24.86, *p* = 0.0014; start vs goal: *t*_(7)_ = 4.564, *p* = 0.026; start vs mid: *t*_(7)_ = 5.555, *p* = 0.0009) (Fig. 2*e*). There was also a trade-off between the speed of decision-making and the safety of choice. Avoid decisions leading to safety were slower (i.e., more difficult) than approach decisions (Fig. 2*f*) (*t*_(7)_ = 6.395, *p* = 0.0004).

### Cognitive modeling of choice under conflict

These decision-making dynamics (log normal RT distributions, RTs with linear coefficient of variation, and trade-offs between decision speed and decision outcome) are shared with speeded choice in human (Forstmann et al., 2016) and nonhuman primates (Shadlen and Shohamy, 2016). They can be explained by sequential sampling models that decompose choice into its latent cognitive mechanisms (Wagenmakers and Brown, 2007; Brown and Heathcote, 2008; Forstmann et al., 2016; Ratcliff et al., 2016). Here, learned sources of reward and punishment are sampled from the environment and memory until an evidence threshold is reached and an approach or avoid choice is made.

The LBA (Brown and Heathcote, 2008) is one such sequential sampling model that permits a complete analytic solution for choices between any number of alternatives (Fig. 2*g*). We used Bayesian estimation via HMCMC to fit the LBA to each animal's approach–avoid decisions from Experiment 1 and derive estimates of three decision-making parameters for each mouse: the rate of evidence accumulation for an approach decision (i.e., *v*_1_, salience of reward), the rate of evidence accumulation for an avoid decision (i.e., *v*_2_, salience of danger), and the threshold of evidence required to reach a decision (i.e., response or decision caution).

The LBA fit the behavioral data well, explaining both RT distributions and choice outcomes (Extended Data Fig. 2-1). LBA parameter estimation for each mouse across the three zones (start, mid, goal) showed that evidence accumulation and decision caution varied dynamically across the track. There was a (3) (zone) × (2) (reward vs danger salience) interaction (*F*_(1,7)_ = 84.159, *p* = 0.00001). Reward salience increased across the track (Mid: *t*_(7)_ = 8.134, *p* < 0.0001 vs avoid) but decreased significantly at the goal zone (*t*_(7)_ = −5.555, *p* = 0.0005 vs avoid) (Fig. 2*h*), explaining the switch from approach to avoid decisions at the goal zone. There was also a significant increase in decision caution as mice approached the goal zone (*F*_(1,7)_ = 35.544, *p* = 0.001) (Fig. 2*i*), showing that more evidence was required to reach a decision as danger loomed, explaining the increased RTs near the goal.

### PVT but not vSub tracks approach–avoidance decisions

**Figure 3.**
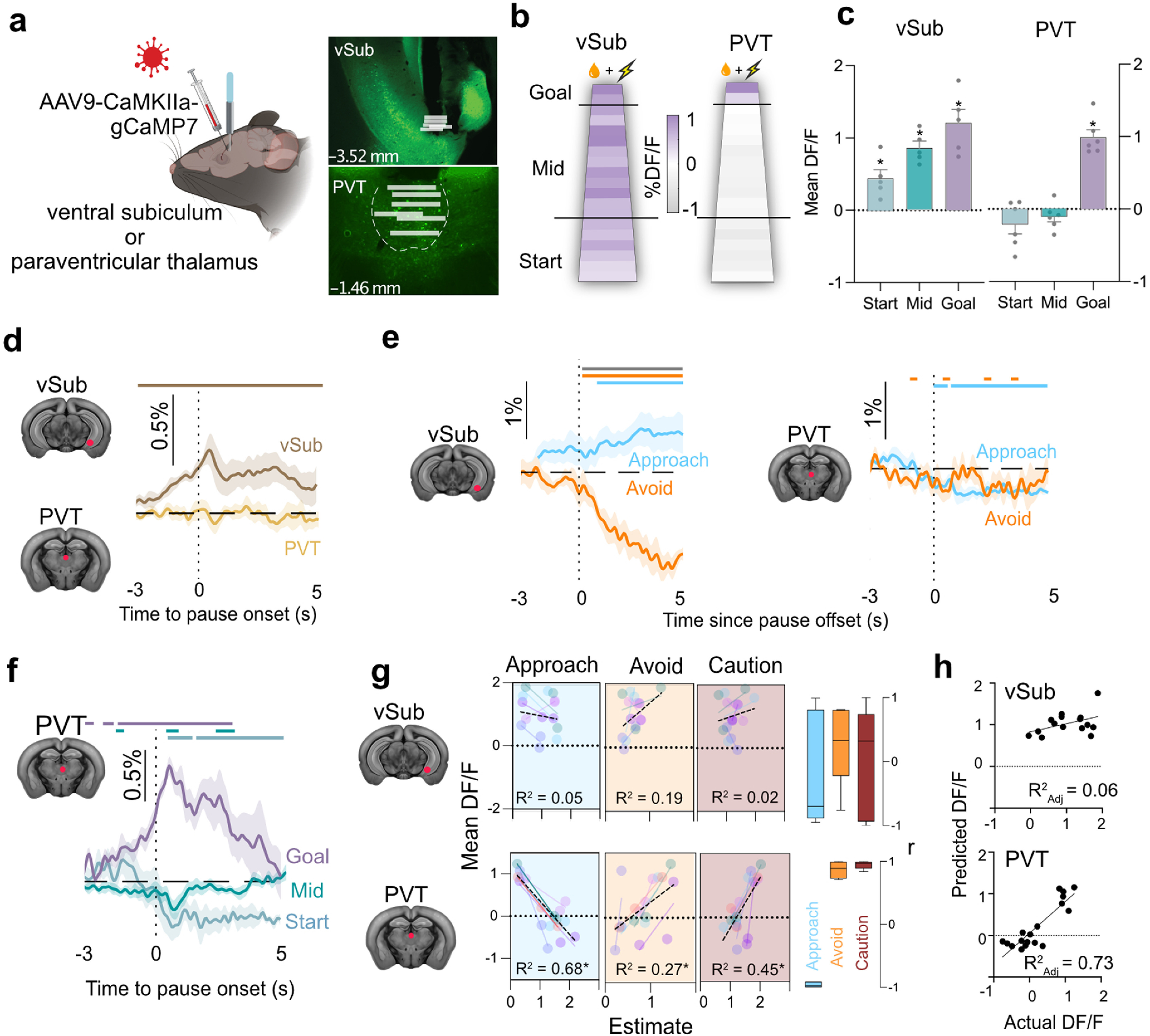
PVT and vSub dynamics during conflict. ***a***, AAV encoding gCaMP7F targeted to PVT or vSub. Fiber optic cannula implanted above injection site. Representative gCaMP7 expression and all fiber optic tip locations (white bars) in vSub and PVT. Distances in millimeters from bregma. Illustration from www.biorender.com. ***b***, Mean %DF/F across the track in 5 cm blocks during conflict (PVT *n* = 6, vSub *n* = 5). ***c***, Mean and SEM %DF/F for start, mid, and goal zone locations (excluding start and Goal boxes). ***d***, Mean and SEM %DF/F at pause onset (0 s) across the track. Colored bars represent periods when 95% CI does not include 0% DF/F. ***e***, Mean and SEM %DF/F at pause offset (0 s) by decision outcome at Start zone. ***f***, PVT mean and SEM %DF/F at pause onsets by location. Colored bars represent periods when 95% CI does not include 0% DF/F. Gray bar represents significant difference between approach versus avoid via 95% CI. ***g***, Relationship between LBA parameters for approach (reward salience), avoid (danger salience), response caution, and mean %DF/F during pauses across track zones fitted by individual mouse (colored points and lines) and overall (black dotted line) with overall *R*^2^. Right, Box plots of individual mouse correlation coefficients between LBA parameters and DF/F in vSub and PVT. ***h***, Linear model of PVT Ca^2+^ transients from LBA parameters. *p < 0.05.

Two brain regions, the vSub, located in the temporal lobe (Gray, 1982; Gray and McNaughton, 2000; Ito and Lee, 2016; Marchant et al., 2016; Çavdaroğlu et al., 2021), and the PVT, located in the midline thalamus (Choi and McNally, 2017; Zhu et al., 2018; Choi et al., 2019; Engelke et al., 2021; Ma et al., 2021), have been implicated in motivational conflict, and so could contribute to these decision-making dynamics. Yet how activity in these brain regions relates to approach–avoidance decision-making remains poorly understood. To address this, in Experiment 2, we infused an AAV encoding the calcium (Ca^2+^) sensor gCaMP7 (Dana et al., 2019) into the vSub or PVT and implanted a fiber optic cannula above these regions. We then used fiber photometry to record vSub (*n* = 5) or PVT (*n* = 6) population Ca^2+^ signals of mice during decision-making under conflict (Fig. 3*a*). We assessed the spatial profile of these Ca^2+^ signals across the track and the relationship between these signals and individual approach–avoid decisions. Fiber tips were located in PVT or vSub, but gCaMP expression extended beyond these regions. Nonetheless, given the presence of strong fluorescence immediately ventral to the fiber tips, we expect the PVT and vSub to have the greatest contribution to the signals recorded.

vSub DF/F was observed across the track (Fig. 3*b*), with DF/F increasing as mice approached the dangerous goal (Fig. 3*b*,*c*) (repeated-measures ANOVA *F*_(2,12)_ = 7.51 *p* = 0.0077; vs 0% DF/F - start: *t*_(4)_ = 3.620 *p* = 0.0224; mid *t*_(4)_ = 9.147 *p* = 0.0008; goal *t*_(4)_ = 6.413 *p* = 0.0030). vSub DF/F also increased before and during individual approach–avoid decisions across the track (Fig. 3*d*; 95% CIs). vSub DF/F in start zone depended on choice outcome, remaining elevated if mice chose to approach the dangerous goal but not if they chose avoidance to the safety of the Start box (Fig. 2*e*; 95% CIs).

In contrast, PVT DF/F showed a spatial bias, with significant increases in DF/F only at the goal zone (Fig. 3*b*,*c*) (repeated-measures ANOVA, *F*_(2,15)_ = 40.62, *p* < 0.0001; vs 0% DF/F - start: *t*_(5)_ = 1.645, *p* = 0.1608; mid *t*_(5)_ = 1.397, *p* = 0.2212; goal *t*_(5)_ = 9.722, *p* = 0.002). PVT DF/F was unrelated to individual approach–avoid decisions if spatial location was ignored (Fig. 3*d*; 95% CIs). Unlike vSub, PVT DF/F after choices in the start zone did not differentiate between approach decisions to stay on the track versus avoid decisions to return to the safety of the Start box (Fig. 3*e*; 95% CIs). Instead, PVT DF/F selectively increased during approach–avoid decisions at the goal zone (Fig. 3*f*; 95% CIs) but not elsewhere on the track, and there were significant reductions in DF/F during approach–avoid decisions at the start zone.

How do vSub and PVT neural dynamics relate to approach–avoid decision-making dynamics? To answer this, we first used HMCMC to derive LBA decision-making parameters (reward salience, danger salience, decision caution) for each mouse during approach–avoid decisions across the three track zones (start zone, mid zone, goal zone), and then we correlated these LBA decision-making parameters for each mouse with their respective vSub and PVT DF/F during approach–avoid decisions. We found that vSub DF/F was unrelated to the components of approach–avoid decisions (Fig. 3*g*) (all *R*^2^ < 0.20, all *p* > 0.05). In contrast, there were strong fits between PVT DF/F and each LBA decision-making parameter (Fig. 3*g*) (approach *R*^2^ = 0.68, *F*_(1,16)_ = 33.59, *p* = 0.0001; avoid *R*^2^ = 0.28, *F*_(1,16)_ = 5.99, *p* = 0.0263; caution *R*^2^ = 0.45, *F*_(1,16)_ = 13.32, *p* = 0.0022). Changes in PVT Ca^2+^ dynamics were most strongly associated with the increases in decision caution and reductions in reward salience as animals approached the goal. Moreover, these LBA decision-making dynamics could be applied within a regression model to accurately predict PVT DF/F across the track (Fig. 3*h*) (β approach = –0.69, β avoid = 0.46, *R*^2^_Adj_ = 0.73, *F*_(2,15)_ = 23.90, *p* = 0.0001).

### A prelimbic → PVT pathway controls decision caution

These findings show that PVT Ca^2+^ dynamics closely track approach–avoidance decisions during motivational conflict but not how PVT contributes to these decisions. PVT DF/F covaried most strongly with dynamic reductions in reward salience and with increases in decision caution as mice approached the dangerous goal. So, PVT could contribute to these changes in reward salience (Zhu et al., 2018; Campus et al., 2019), response caution, or both. The PL is critical for approach–avoid decision-making (Verharen et al., 2019; Kyriazi et al., 2020; Fernandez-Leon et al., 2022) and a major source of excitatory glutamatergic inputs driving PVT neuronal activity (Vertes, 2002; Li and Kirouac, 2012; Otis et al., 2019). We hypothesized that the role of PVT likely depended on this prefrontal input.

**Figure 4.**
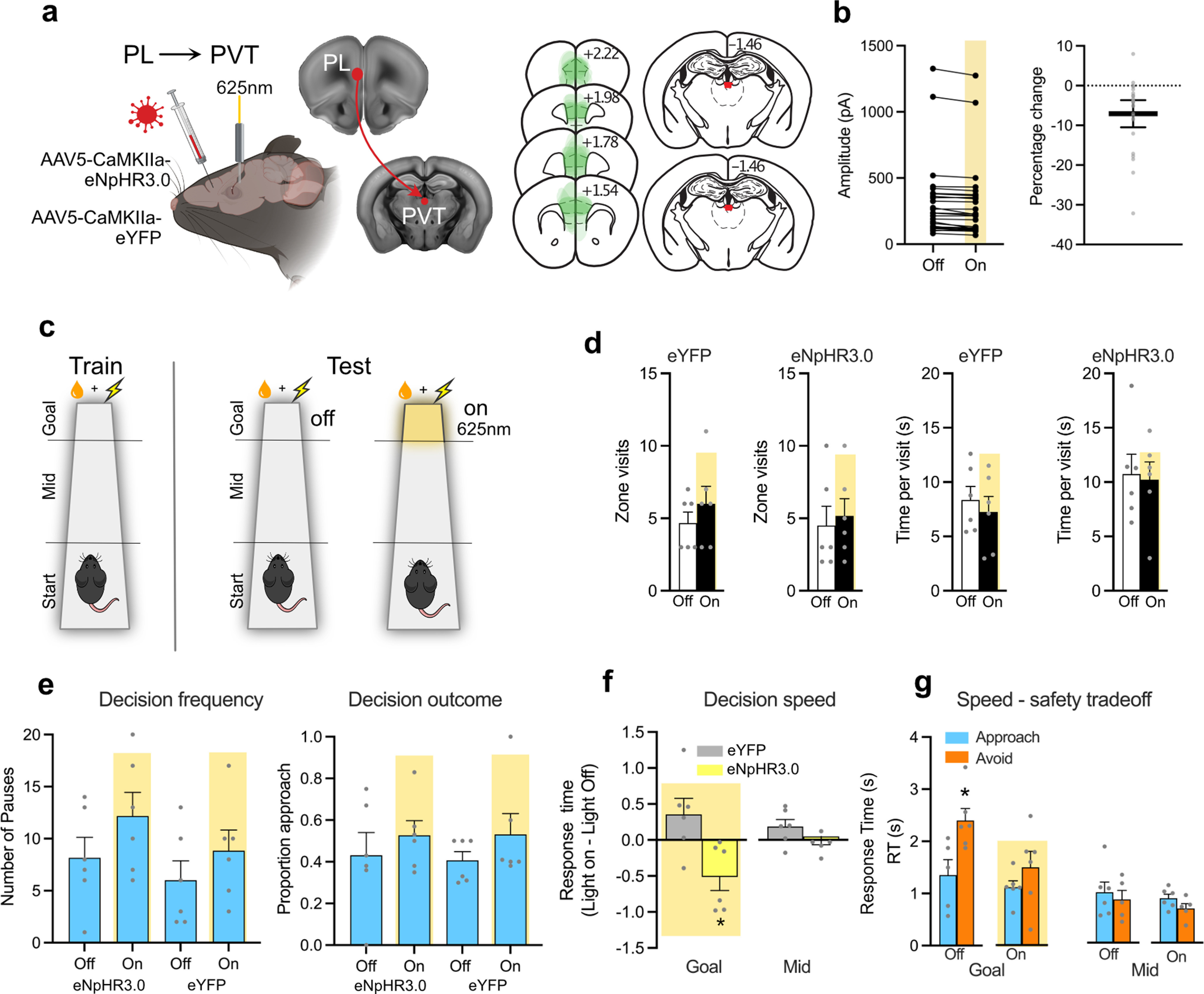
A corticothalamic pathway controls response caution. ***a***, AAV encoding the inhibitory halorhodopsin (eNpHR3.0 *n* = 6) or eYFP (*n* = 6) was targeted to PL and a fiber optic cannula implanted above PVT. AAV expression (each mouse is shown at 15% opacity) in PFC and fiber optic tip location in PVT. Distances in millimeters from bregma. Illustration from www.biorender.com. ***b***, Electrical stimulation of the PL→PVT pathway evoked postsynaptic currents in PVT neurons that were significantly reduced by photoinhibition of PL terminals in the PVT. ***c***, Mice were tested under conflict in the absence (Off) or presence of photoinhibition (On), with photoinhibition delivered only at the goal zone. ***d***, Mean and SEM number of visits to goal zone on the track and duration of stay per visit. ***e***, Mean and SEM decision frequency and outcome at goal zone on tests with (On) and without (Off) photoinhibition. ***f***, Mean and SEM RTs at goal and mid zones. ***g***, Mean and SEM RTs at goal and mid zones for eNpHR3.0 mice on test without (Off) and with (On) photoinhibition. *p < 0.05.

First, we confirmed that we could photoinhibit the PL → PVT pathway. In Experiment 3, we expressed an AAV encoding the inhibitory opsin halorhodopsin (eNpHR3.0) in the PL (Fig. 4*a*) and made whole-cell recordings from PVT neurons (*N* = 26) while evoking EPSCs using a stimulating electrode positioned above fluorescent PL fibers located around the anterior edge of the PVT. Electrical stimulation is not selective for PL fibers, and the PL is one of several brain regions providing excitatory input to PVT neurons, but we could evoke EPSCs in PVT neurons. Photostimulation reduced these EPSCs (Amplitude [pA] – Off vs On: *t*_(25)_ = 4.990, *p* < 0.0001; percent change in amplitude – single mean *t* test vs 0%: *t*_(25)_ = 4.270, *p* = 0.0002), confirming the efficacy of photoinhibition of PL → PVT terminals (Fig. 4*b*). The magnitude of the reduction was modest but consistent with the presumed fractional contribution of PL fibers to the nonselectively electrically evoked current in PVT neurons.

Next, in Experiment 4, we infused an AAV encoding eNpHR3.0 (*n* = 6) or the control eYFP (*n* = 6) into PL (Fig. 4*a*). We implanted an optic fiber above PVT, allowing us to photoinhibit the PL→PVT pathway. After reward training and a single day of conflict training, mice were tested twice under conflict, once in the absence of photoinhibition (Off) and once when the PL→PVT pathway was inhibited via 625 nm light (On) (Fig. 4*c*). We photoinhibited the PL→PVT pathway only when mice were at the goal zone, not elsewhere on the track, because fiber photometry had shown that the goal zone was the only location with significant increases in PVT Ca^2+^ transients. The 625 nm light was triggered by mouse entry to the goal zone, and the light remained on for the duration of the visit. If increases in PVT activity are important for dynamic reductions in reward salience, then inhibition should prevent these reductions and bias approach–avoid decisions toward approach at the goal zone. In contrast, if increases in PVT activity are important for dynamic increases in decision caution as danger looms, then inhibition should prevent this increase in caution and reduce decision speeds at the goal zone.

Inhibiting the PL→PVT pathway had no effect on the number of visits to the goal zone (eNpHR3.0 *t*_(5)_ = 1.512, *p* = 0.191; eYFP *t*_(5)_ = 1.085, *p* = 0.327) or on the time that mice spent there (eNpHR3.0 *t*_(5)_ = 0.2373, *p* = 0.8218; eYFP *t*_(5)_ = 1.080, *p* = 0.3294) (Fig. 4*d*), indicating that photoinhibition did not have obvious nonspecific effects on behavior. PL→PVT photoinhibition had no effect on the outcome of approach–avoid decisions in the goal zone (Group [eNpHR3.0 vs eYFP] main effect: *F*_(1,10)_ = 0.010, *p* = 0.922; Light [On, Off] main effect: *F*_(1,10)_ = 2.976, *p* = 0.115; Group [eNpHR3.0 vs eYFP] × Light [On vs Off] interaction: *F*_(1,10)_ = 0.055 *p* = 0.819) (Fig. 4*e*). PL→PVT photoinhibition also had no effect on the frequency of approach–avoid decisions in the goal zone (Group [eNpHR3.0 vs eYFP] main effect: *F*_(1,10)_ = 1.323, *p* = 0.277; Light [On, Off] main effect: *F*_(1,10)_ = 4.626, *p* = 0.057; Group [eNpHR3.0 vs eYFP] × Light [On vs Off] interaction: *F*_(1,10)_ = 0.135 *p* = 0.721) (Fig. 4*e*). These findings show that PVT did not control dynamic alterations in reward or danger salience here (Zhu et al., 2018; Campus et al., 2019).

Instead, inhibiting the PL→PVT pathway hastened decision speeds in the goal zone (Fig. 4*f*) (Group [eNpHR3.0 vs eYFP] main effect: *F*_(1,10)_ = 1.661, *p* = 0.226; Light [On, Off] main effect: *F*_(1,10)_ = 0.300, *p* = 0.596; Group [eNpHR3.0 vs eYFP] × Light [On v Off] interaction: *F*_(1,10)_ = 8.862 *p* = 0.014). This hastening of decision speeds was specific to the track location where photoinhibition occurred because there was no effect on decision speeds in the mid zone (Group [eNpHR3.0 vs eYFP] main effect: *F*_(1,10)_ = 0.901, *p* = 0.365; Light [On, Off] main effect: *F*_(1,10)_ = 0.756, *p* = 0.405; Group [eNpHR3.0 vs eYFP] × Light [On vs Off] interaction: *F*_(1,10)_ = 0.046 *p* = 0.833). Photoinhibition hastened decision speeds in the eNpHR3.0 group specifically because it abolished the trade-off between decision speed and safety when mice were making approach–avoid decisions (Fig. 4*g*) (Light Off Approach vs Avoid Wilcoxon T = −2.023 *p* = 0.043; Light On: Wilcoxon T = −0.943, *p* = 0.345). This was specific to the track location where photoinhibition occurred because there was no effect on approach versus avoid decision speeds in the mid zone (Light Off Approach vs Avoid Wilcoxon T = 0.677, *p* = 0.498; Light On: Wilcoxon T = −0.105, *p* = 0.917).

## Discussion

Decisions about whether to approach or avoid a food source while under the threat of danger and predation require balancing the competing demands of speed in decision-making with safety of choice. Premature decisions risk injury or death, whereas failures to decide in a timely manner risk goal loss. Here we studied how mice make these approach–avoid decisions. We show dynamic changes in approach–avoidance decisions that depend on both the proximity of danger and choice outcome. Most importantly, we show that mice trade off decision-speed for decision safety when making approach–avoid decisions. We show that this trade-off between decision speed and decision safety is linked to a corticothalamic pathway from the PL to the PVT that dynamically adjusts decision caution as danger nears.

### Approach–avoid decision-making under motivational conflict

Our findings show that decision-making under fundamental survival conditions in mice shares the same lawful features as speeded decision-making in human (Forstmann et al., 2016) and nonhuman primates (Shadlen and Shohamy, 2016). Sequential sampling is a highly efficient, domain-general solution to the problem of fast, accurate decision-making that uses common mechanisms to explain decision outcome and decision time (Gold and Shadlen, 2002; Gluth et al., 2015; Forstmann et al., 2016; Shadlen and Shohamy, 2016; Bakkour et al., 2019). We show that decision-making during motivational conflict in mice has the hallmarks of a sequential sampling mechanism that is shared by human decision-making during word versus nonword recognition (Forstmann et al., 2016), visual hallucinations (Pearson and Brascamp, 2008), and in nonhuman primate motion perception (Shadlen and Newsome, 2001), among others.

Specifically, the spatial and temporal dynamics of behavior were well explained by the LBA model, an exemplar sequential sampling model (Donkin et al., 2011a, b). Formal cognitive modeling showed that there were dynamic changes in the salience of reward and danger across space, with reductions in reward salience as danger loomed. There were also strategic adjustments in response caution across space. Most caution was exercised closest to the goal, leading to longer decision times at the goal. Together, these dynamic changes in decision-making generated a bistable phenotype with mice oscillating between the relative safety of the Start box and the relative danger of the goal, and trading off speed in decision-making for safety of choice.

These findings underscore the utility of computational process models of choice, such as the LBA, to understanding motivated behavior. These models have been used to study neural correlates of sensory decision-making in nonhuman primates (e.g., Shadlen and Newsome, 1996; Gold and Shadlen, 2007; Brody and Hanks, 2016; Shadlen and Shohamy, 2016) but are yet to see widespread application to problems in motivation (Walters et al., 2019; McNally, 2021). Key advantages of this approach are that it allows a rich examination of behavior because of joint modeling of RTs as well as choice outcome and that it makes specific predictions about the structure of underlying neural processes (Johnson and Ratcliff, 2018). However, although our findings highlight the utility of these models to explaining behavior under motivational conflict, much remains to be learned. For example, we studied only male mice, so the nature and role of sex differences in approach–avoidance decision-making remain to be determined. The modeling approach taken here may be useful in identifying sex differences in underlying choice mechanics not otherwise obvious from behavior. In addition, although we show that these models can successfully describe behavior under relatively simple and predictable conditions, it remains unclear whether these models can explain behavior under more complex, less predictable conditions. Under complex conditions, other more sophisticated deliberation mechanisms involving memory exploration and information search may supplement or replace the processes described here (Redish, 2016; Walters et al., 2019; Hunt et al., 2021).

Regardless, many behavioral tasks assessing choice in the laboratory under notionally Pavlovian or instrumental conditions (e.g., Pavlovian to instrumental transfer; sign tracking vs goal tracking; natural vs drug reward; social vs drug reward) are conducted under conditions similar to those used here. The focus in these tasks is often on how environmental or neural manipulations affect choice outcome. However, as shown here, choice behavior even under simple conditions is richer than simply what is chosen. It can involve biases, dynamic changes in decision speed, and trade-offs between decision speed and decision outcome. The mechanisms for such choices could differ across tasks (e.g., Pavlovian vs instrumental), but the application of formal cognitive models to behavior in these tasks to jointly model choice outcome and RTs may provide a useful addition to traditional associative approaches in identifying computational similarities in information processing and their underlying circuit mechanisms across these distinct tasks (Redish et al., 2022).

### Role of the corticothalamic pathway and vSub in motivational conflict

A top-down corticothalamic pathway controls strategic adjustments in decision caution during decision-making under motivational conflict. Strategic adjustments in decision or response caution are among the most elementary forms of cognitive control. We show here that increases in PVT activity, as inferred from increases in PVT Ca^2+^ transients, were selectively observed during approach–avoid decisions at the goal. There were no increases in PVT Ca^2+^ transients during the same behaviors at other locations on the track. Cognitive modeling indicated that these increases in PVT Ca^2+^ transients reflected an increase in the amount of evidence required to reach a decision, thereby increasing the time taken to choose between approaching or avoiding the dangerous goal. This increased evidence threshold drives a trade-off between the speed of choice and the safety of choice. Consistent with these model predictions, inhibiting the PL → PVT pathway prevented the increase in decision times at the dangerous goal and abolished the speed–safety trade-off.

In humans, neuroimaging studies link the trade-off between speed and accuracy in perceptual decision-making to corticostriatal circuits (Forstmann et al., 2008, 2010; Bogacz et al., 2010; Winkel et al., 2016), but ensemble recordings in rodent striatum have not consistently identified similar signals (Stott and Redish, 2014; Brody and Hanks, 2016). Instead, corticostriatal circuits have well-established roles in integrating stimulus value with action information to control value-based choice (Balleine and O'Doherty, 2010; Hannah and Aron, 2021; Weglage et al., 2021; Tang et al., 2022). The role we identify for PVT in decision caution is complementary to these corticostriatal choice mechanisms. PVT neurons have extensive, highly collateralized projections to ventral corticostriatal circuits (Dong et al., 2017). So, one possibility is that PVT broadcasts an evidence threshold across ventral corticostriatal value networks to control the speed of value-based decision-making.

The role of vSub in these processes is less clear. vSub is implicated in behavior under motivational conflict (Ito and Lee, 2016; Marchant et al., 2016; Çavdaroğlu et al., 2021). However, the role of vSub in conflict behavior here was distinct to that of PVT. Whereas PVT showed spatially and behaviorally selective increases in Ca^2+^ DF/F, vSub Ca^2+^ DF/F was elevated nonselectively across the track, including modest elevations during choices. Unlike PVT, there was no significant relationship between vSub Ca^2+^ DF/F and individual decision-making parameters. So, we consider it unlikely that vSub contributes directly to approach or avoid decisions. Instead, vSub may be linked to anxiety and arousal in this task. Gray and McNaughton (Gray, 1982; Gray and McNaughton, 2000) argued that approach–avoidance conflict recruits a behavioral inhibition system localized to the septohippocampal system. The behavioral inhibition system supports changes in arousal and attention. The behavioral inhibition system remains recruited while conflict persists but not when avoidance behavior successfully removes the animal from the conflict situation. The profile of vSub DF/F reported here, increasing with proximity to the goal and decreasing after choices to avoid to the safety of home, is consistent with this (Gray, 1982; Gray and McNaughton, 2000).

In conclusion, our findings demonstrate that a discrete brain circuit involving the PVT and its prefrontal cortical input dynamically adjusts decision caution during motivational conflict, trading off decision speed for decision safety when danger is close. They identify the corticothalamic pathway as central to cognitive control during decision-making under conflict. PVT has been implicated in a variety of motivated behaviors, but a general account of its function remains elusive (Kirouac, 2015). Many PVT-dependent tasks involve choices between different, incompatible behaviors (McNally, 2021). These include choices between approach and avoid (Choi and McNally, 2017; Choi et al., 2019; Engelke et al., 2021), between different defensive behaviors (e.g., fight vs flight) (Ma et al., 2021), between approaching different sources of reward (e.g., sign vs goal tracking, Pavlovian to instrumental transfer) (Campus et al., 2019), and between persisting with or ceasing a behavior that no longer yields reward (Hamlin et al., 2009; Otis et al., 2017). Like the approach–avoidance choices studied here, these choices necessitate trade-off between the speed and outcome of decision-making. Our finding that PVT controls this trade-off by determining the amount of caution exercised in making a choice provides a mechanism for cognitive control applicable across a range of behaviors and tasks. Moreover, it identifies PVT and its prefrontal cortical input as targets to understand and remediate the deficits in decision caution characteristic of unsafe or impulsive choices.

## References

[B1] Annis J, Miller BJ, Palmeri TJ (2017) Bayesian inference with Stan: a tutorial on adding custom distributions. Behav Res Methods 49:863–886. 10.3758/s13428-016-0746-9 27287444 PMC5149118

[B2] Bach DR, Dayan P (2017) Algorithms for survival: a comparative perspective on emotions. Nat Rev Neurosci 18:311–319. 10.1038/nrn.2017.35 28360419

[B3] Bach DR, Guitart-Masip M, Packard PA, Miro J, Falip M, Fuentemilla L, Dolan RJ (2014) Human hippocampus arbitrates approach–avoidance conflict. Curr Biol 24:541–547. 10.1016/j.cub.2014.01.046 24560572 PMC3969259

[B4] Bakkour A, Palombo DJ, Zylberberg A, Kang YH, Reid A, Verfaellie M, Shadlen MN, Shohamy D (2019) The hippocampus supports deliberation during value-based decisions. Elife 8:e46080. 10.7554/Elife.46080 31268419 PMC6693920

[B5] Balleine BW, O'Doherty JP (2010) Human and rodent homologies in action control: corticostriatal determinants of goal-directed and habitual action. Neuropsychopharmacology 35:48–69. 10.1038/npp.2009.131 19776734 PMC3055420

[B6] Bogacz R, Wagenmakers EJ, Forstmann BU, Nieuwenhuis S (2010) The neural basis of the speed-accuracy tradeoff. Trends Neurosci 33:10–16. 10.1016/j.tins.2009.09.002 19819033

[B7] Brody CD, Hanks TD (2016) Neural underpinnings of the evidence accumulator. Curr Opin Neurobiol 37:149–157. 10.1016/j.conb.2016.01.003 26878969 PMC5777584

[B8] Brown SD, Heathcote A (2008) The simplest complete model of choice response time: linear ballistic accumulation. Cogn Psychol 57:153–178. 10.1016/j.cogpsych.2007.12.002 18243170

[B9] Campus P, Covelo IR, Kim Y, Parsegian A, Kuhn BN, Lopez SA, Neumaier JF, Ferguson SM, Solberg Woods LC, Sarter M, Flagel SB (2019) The paraventricular thalamus is a critical mediator of top-down control of cue-motivated behavior in rats. Elife 8:e49041. 10.7554/Elife.49041 31502538 PMC6739869

[B10] Çavdaroğlu B, Riaz S, Yeung EH, Lee AC, Ito R (2021) The ventral hippocampus is necessary for cue-elicited, but not outcome driven approach–avoidance conflict decisions: a novel operant choice decision-making task. Neuropsychopharmacology 46:632–642. 10.1038/s41386-020-00898-z 33154580 PMC8027851

[B11] Choi EA, McNally GP (2017) Paraventricular thalamus balances danger and reward. J Neurosci 37:3018–3029. 10.1523/JNEUROSCI.3320-16.2017 28193686 PMC6596734

[B12] Choi EA, Jean Richard dit Bressel P, Clifford CW, McNally GP (2019) Paraventricular thalamus controls behavior during motivational conflict. J Neurosci 39:4945–4958. 10.1523/JNEUROSCI.2480-18.2019 30979815 PMC6670259

[B13] Dana H, Sun Y, Mohar B, Hulse BK, Kerlin AM, Hasseman JP, Tsegaye G, Tsang A, Wong A, Patel R, Macklin JJ, Chen Y, Konnerth A, Jayaraman V, Looger LL, Schreiter ER, Svoboda K, Kim DS (2019) High-performance calcium sensors for imaging activity in neuronal populations and microcompartments. Nat Methods 16:649–657. 10.1038/s41592-019-0435-6 31209382

[B14] Dong X, Li S, Kirouac GJ (2017) Collateralization of projections from the paraventricular nucleus of the thalamus to the nucleus accumbens, bed nucleus of the stria terminalis, and central nucleus of the amygdala. Brain Struct Funct 222:3927–3943. 10.1007/s00429-017-1445-8 28528379

[B15] Donkin C, Brown S, Heathcote A (2011a) Drawing conclusions from choice response time models: a tutorial using the linear ballistic accumulator. J Math Psychol 55:140–151. 10.1016/j.jmp.2010.10.001

[B16] Donkin C, Brown S, Heathcote A, Wagenmakers EJ (2011b) Diffusion versus linear ballistic accumulation: different models but the same conclusions about psychological processes? Psychon Bull Rev 18:61–69. 10.3758/s13423-010-0022-4 21327360 PMC3042112

[B17] Engelke DS, Zhang XO, O'Malley JJ, Fernandez-Leon JA, Li S, Kirouac GJ, Beierlein M, Do-Monte FH (2021) A hypothalamic-thalamostriatal circuit that controls approach–avoidance conflict in rats. Nat Commun 12:2517. 10.1038/s41467-021-22730-y 33947849 PMC8097010

[B18] Fernandez-Leon JA, Engelke DS, Aquino-Miranda G, Goodson A, Rasheed MN, Do-Monte FH (2022) Neural correlates and determinants of approach–avoidance conflict in the prelimbic prefrontal cortex. Elife 10:e74950. 10.7554/eLife.74950 PMC8853658 34913438

[B19] Forstmann BU, Dutilh G, Brown S, Neumann J, von Cramon DY, Ridderinkhof KR, Wagenmakers EJ (2008) Striatum and pre-SMA facilitate decision-making under time pressure. Proc Natl Acad Sci USA 105:17538–17542. 10.1073/pnas.0805903105 18981414 PMC2582260

[B20] Forstmann BU, Anwander A, Schafer A, Neumann J, Brown S, Wagenmakers EJ, Bogacz R, Turner R (2010) Cortico-striatal connections predict control over speed and accuracy in perceptual decision making. Proc Natl Acad Sci USA 107:15916–15920. 10.1073/pnas.1004932107 20733082 PMC2936628

[B21] Forstmann BU, Ratcliff R, Wagenmakers EJ (2016) Sequential sampling models in cognitive neuroscience: advantages, applications, and extensions. Annu Rev Psychol 67:641–666. 10.1146/annurev-psych-122414-033645 26393872 PMC5112760

[B22] Gelman A, Rubin DB (1992) Inference from iterative simulation using multiple sequences. Statist Sci 7:457–511. 10.1214/ss/1177011136

[B23] Gelman A, Carlin JB, Stern HS, Dunson DB, Vehtari A, Rubin DB (2013) Bayesian data analysis, Vol 3. Boca Raton, FL: Chapman and Hall/CRC.

[B24] Gluth S, Sommer T, Rieskamp J, Buchel C (2015) Effective connectivity between hippocampus and ventromedial prefrontal cortex controls preferential choices from memory. Neuron 86:1078–1090. 10.1016/j.neuron.2015.04.023 25996135

[B25] Gold JI, Shadlen MN (2002) Banburismus and the brain: decoding the relationships between sensory stimuli, decisions, and reward. Neuron 36:299–308. 10.1016/S0896-6273(02)00971-6 12383783

[B26] Gold JI, Shadlen MN (2007) The neural basis of decision making. Annu Rev Neurosci 30:535–574. 10.1146/annurev.neuro.29.051605.113038 17600525

[B27] Gray JA (1982) The neuropsychology of anxiety: an enquiry in to the functions of the septo-hippocampal system. Oxford: Oxford UP.

[B28] Gray JA, McNaughton N (2000) The neuropsychology of anxiety: an enquiry into the functions of the septo-hippocampal system. Oxford: Oxford UP.

[B29] Hamlin AS, Clemens KJ, Choi EA, McNally GP (2009) Paraventricular thalamus mediates context-induced reinstatement (renewal) of extinguished reward seeking. Eur J Neurosci 29:802–812. 10.1111/j.1460-9568.2009.06623.x 19200064

[B30] Hannah R, Aron AR (2021) Towards real-world generalizability of a circuit for action-stopping. Nat Rev Neurosci 22:538–552. 10.1038/s41583-021-00485-1 34326532 PMC8972073

[B31] Hoffman MD, Gelman A (2014) The no-U-turn sampler: adaptively setting path lengths in Hamiltonian Monte Carlo. J Machine Learn Res 15:1351–1381.

[B32] Hull CL (1938) The goal-gradient hypothesis applied to some 'field-force' problems in the behavior of young children. Psychol Rev 45:271–298. 10.1037/h0053885

[B33] Hunt LT, Daw ND, Kaanders P, MacIver MA, Mugan U, Procyk E, Redish AD, Russo E, Scholl J, Stachenfeld K, Wilson CR, Kolling N (2021) Formalizing planning and information search in naturalistic decision-making. Nat Neurosci 24:1051–1074. 10.1038/s41593-021-00866-w 34155400

[B34] Ito R, Lee AC (2016) The role of the hippocampus in approach–avoidance conflict decision-making: evidence from rodent and human studies. Behav Brain Res 313:345–357. 10.1016/j.bbr.2016.07.039 27457133

[B35] Jean Richard dit Bressel P, Clifford CW, McNally GP (2020) Analyzing event-related transients: confidence intervals, permutation tests, and consecutive thresholds. Front Mol Neurosci 13:14. 10.3389/fnmol.2020.00014 32116547 PMC7017714

[B36] Johnson EJ, Ratcliff R (2018) Computational and process models of decision making in psychology and behavioral economics. In: Neuroeconomics: decision making and the brain (Glimcher PW, Fehr E, eds), pp 35–47. Amsterdam: Elsevier.

[B37] Kirouac GJ (2015) Placing the paraventricular thalamus within the brain circuits that control behavior. Neurosci Biobehav Rev 56:315–329. 10.1016/j.neubiorev.2015.08.005 26255593

[B38] Kyriazi P, Headley DB, Pare D (2020) Different multidimensional representations across the amygdalo-prefrontal network during an approach–avoidance task. Neuron 107:717–730. 10.1016/j.neuron.2020.05.039 32562662 PMC7442738

[B39] Laming DR (1968) Information theory of choice-reaction times. San Diego: Academic.

[B40] Li S, Kirouac GJ (2012) Sources of inputs to the anterior and posterior aspects of the paraventricular nucleus of the thalamus. Brain Struct Funct 217:257–273. 10.1007/s00429-011-0360-7 22086160

[B41] Ma J, du Hoffmann J, Kindel M, Beas BS, Chudasama Y, Penzo MA (2021) Divergent projections of the paraventricular nucleus of the thalamus mediate the selection of passive and active defensive behaviors. Nat Neurosci 24:1429–1440. 10.1038/s41593-021-00912-7 34413514 PMC8484052

[B42] Marchant NJ, Campbell EJ, Whitaker LR, Harvey BK, Kaganovsky K, Adhikary S, Hope BT, Heins RC, Prisinzano TE, Vardy E, Bonci A, Bossert JM, Shaham Y (2016) Role of ventral subiculum in context-induced relapse to alcohol seeking after punishment-imposed abstinence. J Neurosci 36:3281–3294. 10.1523/JNEUROSCI.4299-15.2016 26985037 PMC4792939

[B43] McNally GP (2021) Motivational competition and the paraventricular thalamus. Neurosci Biobehav Rev 125:193–207. 10.1016/j.neubiorev.2021.02.021 33609570

[B44] Miller NE (1944) Experimental studies of conflict. Pers Behav Disord 431–465.

[B45] Mobbs D, Headley DB, Ding W, Dayan P (2020) Space, time, and fear: survival computations along defensive circuits. Trends Cogn Sci 24:228–241. 10.1016/j.tics.2019.12.016 32029360

[B46] Nguyen D, Schumacher A, Erb S, Ito R (2015) Aberrant approach–avoidance conflict resolution following repeated cocaine pre-exposure. Psychopharmacology (Berl) 232:3573–3583. 10.1007/s00213-015-4006-y 26156635

[B47] O'Neil EB, Newsome RN, Li IH, Thavabalasingam S, Ito R, Lee AC (2015) Examining the role of the human hippocampus in approach–avoidance decision making using a novel conflict paradigm and multivariate functional magnetic resonance imaging. J Neurosci 35:15039–15049. 10.1523/JNEUROSCI.1915-15.2015 26558775 PMC6605357

[B48] Otis JM, Namboodiri VM, Matan AM, Voets ES, Mohorn EP, Kosyk O, McHenry JA, Robinson JE, Resendez SL, Rossi MA, Stuber GD (2017) Prefrontal cortex output circuits guide reward seeking through divergent cue encoding. Nature 543:103–107. 10.1038/nature21376 28225752 PMC5772935

[B49] Otis JM, Zhu MH, Namboodiri VM, Cook CA, Kosyk O, Matan AM, Ying R, Hashikawa Y, Hashikawa K, Trujillo-Pisanty I, Guo J, Ung RL, Rodriguez-Romaguera J, Anton ES, Stuber GD (2019) Paraventricular thalamus projection neurons integrate cortical and hypothalamic signals for cue-reward processing. Neuron 103:423–429. 10.1016/j.neuron.2019.05.018 31196673 PMC6773659

[B50] Paxinos G, Watson C (2019) The mouse brain in stereotaxic coordinates, Ed 5. San Diego: Academic.

[B51] Pearson J, Brascamp J (2008) Sensory memory for ambiguous vision. Trends Cogn Sci 12:334–341. 10.1016/j.tics.2008.05.006 18684661

[B52] R Development Core Team (2017) R: a language and environment for statistical computing. Vienna: R Development Core Team.

[B53] Ratcliff R, Smith PL, Brown SD, McKoon G (2016) Diffusion decision model: current issues and history. Trends Cogn Sci 20:260–281. 10.1016/j.tics.2016.01.007 26952739 PMC4928591

[B54] Redish AD (2016) Vicarious trial and error. Nat Rev Neurosci 17:147–159. 10.1038/nrn.2015.30 26891625 PMC5029271

[B55] Redish AD, Kepecs A, Anderson LM, Calvin OL, Grissom NM, Haynos AF, Heilbronner SR, Herman AB, Jacob S, Ma S (2022) Computational validity: using computation to translate behaviours across species. Philos Trans R Soc Lond B Biol Sci 377:20200525. 34957854 10.1098/rstb.2020.0525 PMC8710889

[B56] Schumacher A, Vlassov E, Ito R (2016) The ventral hippocampus, but not the dorsal hippocampus is critical for learned approach–avoidance decision making. Hippocampus 26:530–542. 10.1002/hipo.22542 26493973

[B57] Shadlen MN, Newsome RT (1996) Motion perception: seeing and deciding. Proc Natl Acad Sci USA 93:628–633. 10.1073/pnas.93.2.628 8570606 PMC40102

[B58] Shadlen MN, Newsome RN (2001) Neural basis of a perceptual decision in the parietal cortex (Area LIP) of the Rhesus monkey. J Neurophysiol 86:1916–1936. 10.1152/jn.2001.86.4.1916 11600651

[B59] Shadlen MN, Shohamy D (2016) Decision making and sequential sampling from memory. Neuron 90:927–939. 10.1016/j.neuron.2016.04.036 27253447 PMC4891701

[B60] Stan Development Team (2015) Stan: a C++ library for probability and sampling. Stan Development Team. Available at https://mc-stan.org.

[B61] Stott JJ, Redish AD (2014) A functional difference in information processing between orbitofrontal cortex and ventral striatum during decision-making behaviour. Philos Trans R Soc Lond B Biol Sci 369:20130472. 25267815 10.1098/rstb.2013.0472 PMC4186226

[B62] Tang H, Costa VD, Bartolo R, Averbeck BB (2022) Differential coding of goals and actions in ventral and dorsal corticostriatal circuits during goal-directed behavior. Cell Rep 38:110198. 10.1016/j.celrep.2021.110198 34986350 PMC9608360

[B63] Thompson SM, Berkowitz LE, Clark BJ (2018) Behavioral and neural subsystems of rodent exploration. Learn Motiv 61:3–15. 10.1016/j.lmot.2017.03.009 30270939 PMC6159932

[B64] Verharen JP, van den Heuvel MW, Luijendijk M, Vanderschuren L, Adan RA (2019) Corticolimbic mechanisms of behavioral inhibition under threat of punishment. J Neurosci 39:4353–4364. 10.1523/JNEUROSCI.2814-18.2019 30902868 PMC6538860

[B65] Verharen JP, den Ouden HE, Adan RA, Vanderschuren L (2020) Modulation of value-based decision making behavior by subregions of the rat prefrontal cortex. Psychopharmacology (Berl) 237:1267–1280. 10.1007/s00213-020-05454-7 32025777 PMC7196947

[B66] Vertes RP (2002) Analysis of projections from the medial prefrontal cortex to the thalamus in the rat, with emphasis on nucleus reuniens. J Comp Neurol 442:163–187. 10.1002/cne.10083 11754169

[B67] Wagenmakers EJ, Brown S (2007) On the linear relation between the mean and the standard deviation of a response time distribution. Psychol Rev 114:830–841. 10.1037/0033-295X.114.3.830 17638508

[B68] Walters CJ, Jubran J, Sheehan A, Erickson MT, Redish AD (2019) Avoid-approach conflict behaviors differentially affected by anxiolytics: implications for a computational model of risky decision-making. Psychopharmacology (Berl) 236:2513–2525. 10.1007/s00213-019-05197-0 30863879 PMC6697581

[B69] Weglage M, Wärnberg E, Lazaridis I, Calvigioni D, Tzortzi O, Meletis K (2021) Complete representation of action space and value in all dorsal striatal pathways. Cell Rep 36:109437. 10.1016/j.celrep.2021.109437 34320355

[B70] Winkel J, Hawkins GE, Ivry RB, Brown SD, Cools R, Forstmann BU (2016) Focal striatum lesions impair cautiousness in humans. Cortex 85:37–45. 10.1016/j.cortex.2016.09.023 27810498

[B71] Wu CT, Haggerty D, Kemere C, Ji D (2017) Hippocampal awake replay in fear memory retrieval. Nat Neurosci 20:571–580. 10.1038/nn.4507 28218916 PMC5373994

[B72] Zhu Y, Nachtrab G, Keyes PC, Allen WE, Luo L, Chen X (2018) Dynamic salience processing in paraventricular thalamus gates associative learning. Science 362:423–429. 10.1126/science.aat0481 30361366 PMC6521722

