# Supplementary figures and images for: A Corticothalamic Circuit Trades off Speed for Safety during Decision-Making under Motivational Conflict
Source: J Neurosci. 2022 Apr 20;42(16):3473–83. doi: 10.1523/JNEUROSCI.0088-22.2022 (PMC9034778; doi:10.1523/JNEUROSCI.0088-22.2022)

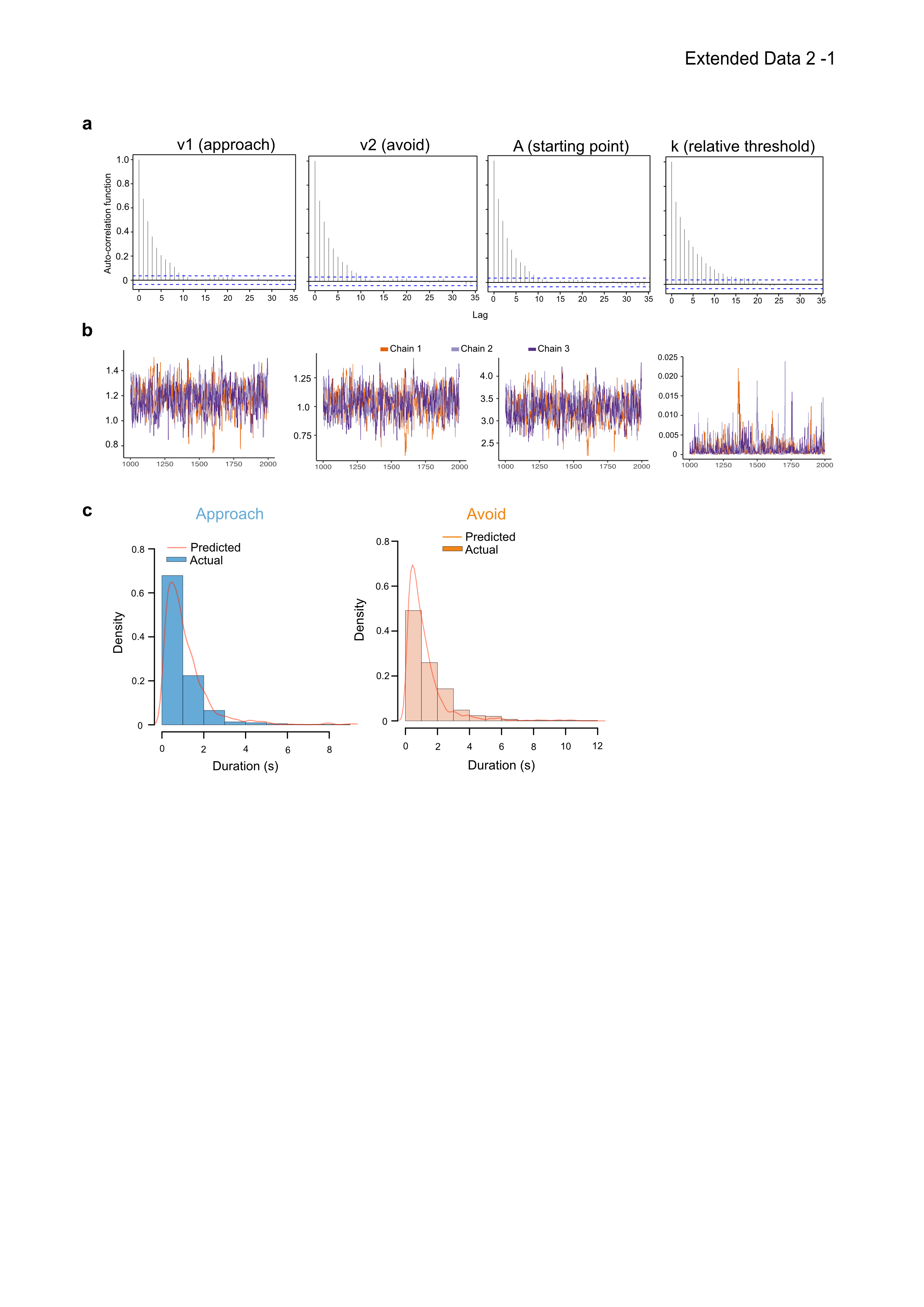

Supplement: Figure 2-1 — Bayesian parameter estimation of LBA via Hamiltonian Markov Chain Monte Carlo. a) Autocorrelation functions for samples returned by Stan. Autocorrelations dropped to zero at around lags of 10, indicating that the sampler efficiently explored the posteriors for each parameter. b) Samples from each chain as a function of iteration showing strong central tendencies, remaining around constant values, with strong overlap between chains, indicating convergence to the posterior distribution. c) Posterior predictive check indicating good fit between the model predictions (line) and the observed (histograms) decision times. Warmup = 1000; iteration = 2000; thinning = 1; delta = 0.8. Three chains were run to evaluate convergence with a Gelman-Rubin's criteria of R ^ < 1.1 and an effective sample size (Neff) > 100. Download Figure 2-1, TIF file. [file ns-JN-RM-0088-22-s01.tif]
